# Sex-Based Differences in Patient-Reported Outcome Measures Are Not Present Three Months After ACL Reconstruction

**DOI:** 10.3390/jcm15020680

**Published:** 2026-01-14

**Authors:** Abdulmajeed Alfayyadh, Jack R. Williams, Kelsey Neal, Ashutosh Khandha, Lynn Snyder-Mackler, Thomas S. Buchanan

**Affiliations:** 1 Department of Physical Therapy and Health Rehabilitation, College of Applied Medical Sciences, Jouf University, Sakaka 72388, Saudi Arabia; 2 Department of Bioengineering, Stanford University, Stanford, CA 94305, USA; jackrw@stanford.edu; 3 School of Medicine, University of New Mexico, Albuquerque, NM 87106, USA; keaneal@salud.unm.edu; 4 Department of Biomedical Engineering, University of Delaware, Newark, DE 19716, USA; ashutosh@udel.edu; 5 Department of Physical Therapy, University of Delaware, Newark, DE 19716, USA; smack@udel.edu; 6 Department of Mechanical Engineering, University of Delaware, Newark, DE 19716, USA; buchanan@udel.edu

**Keywords:** ACL reconstruction, sex-based differences, patient-reported outcomes, KOOS, IKDC, KOS-ADLS, ACL-RSI, TSK

## Abstract

**Background:** Patient-reported outcome measures (PROMs) provide important insights into recovery after anterior cruciate ligament reconstruction (ACLR). Previous research suggests that males and females recover differently after ACLR, with females reporting greater pain, slower functional gains, and lower psychological readiness at later stages of rehabilitation. However, it is unknown if patient-reported outcomes differ by sex early after ACLR. To address this gap, we conducted a cross-sectional analysis comparing patient-reported outcome measures between sexes three months after ACLR. We hypothesized that females would report worse PROMs compared to males. **Methods:** This cross-sectional analysis used data from a prospectively maintained ACL reconstruction cohort. Fifty-six individuals (female: 23 and male: 33) with primary, unilateral ACLR completed PROMs three months after surgery. These PROMs included the Knee Injury and Osteoarthritis Outcome Score (KOOS; Symptoms, Pain, Activities of Daily Living, Sport and Recreation, Quality of Life), International Knee Documentation Committee (IKDC) subjective score, Knee Outcome Survey–Activities of Daily Living Scale (KOS-ADLS), Anterior Cruciate Ligament–Return to Sport After Injury (ACL-RSI), and the Tampa Scale of Kinesiophobia (TSK). All outcomes were expressed on a 0 to 100 percent scale, with higher scores indicating better outcomes, except for TSK, where lower scores indicated better outcomes. Normality was assessed within sex, using the Shapiro–Wilk test. Two-tailed independent-samples *t*-tests with Welch correction were used for approximately normal variables; otherwise, Mann–Whitney U tests were utilized (α = 0.05). Several outcomes had limited statistical power to detect MCID-sized differences, and findings for these measures should be interpreted cautiously. **Results:** No significant differences between sexes were found for any of the PROMs. Males trended towards having better KOOS Sport and Recreation and IKDC, but these were not statistically significant, and the effect sizes were small-to-moderate. **Conclusions:** No statistically significant sex-based differences were detected in PROMs at approximately 3 months after ACLR, indicating that any sex-related divergences between these measures may not occur until later in recovery.

## 1. Introduction

Anterior cruciate ligament reconstruction (ACLR) is performed widely in young, active populations to restore knee stability and facilitate return to sport [1]. Despite surgical advances and structured rehabilitation, recovery trajectories vary. Patient-reported outcome measures (PROMs) capture pain, symptoms, function, sport participation, and knee-related quality of life that matter directly to patients and clinicians. There is evidence that sex-based differences in functional and patient-reported outcomes exist after ACL injury and reconstruction; however, this remains an area of active debate. Elucidating whether postoperative PROMs differ by sex has practical implications for setting expectations, tailoring education, and prioritizing psychosocial or functional interventions post-ACLR. Because recovery after ACLR spans multiple domains, we selected PROMs that capture distinct but related constructs, including knee symptoms and pain (KOOS), perceived knee function during daily and sport activities (IKDC, KOOS Sport and Recreation, KOS-ADLS), knee-related quality of life (KOOS QOL), psychological readiness to return to sport (ACL-RSI), and fear of movement or reinjury (TSK). These instruments are widely used in ACLR research and have established reliability, validity, and responsiveness for monitoring recovery [2,3].

Multiple studies report that, from 9 to 60 months after ACLR, females often score lower than males in knee function and quality of life (QOL) indices, including the International Knee Documentation Committee subjective knee form (IKDC) and the Knee Injury and Osteoarthritis Outcome Score (KOOS) Sport and Recreation and QOL subscales, although the magnitude is generally small and certainty varies [4,5,6]. Longitudinal analyses indicate that divergence in KOOS QOL may emerge over time, with males demonstrating steeper improvement trajectories between 36 and 60 months, while females plateau earlier [6]. Psychological readiness follows a related pattern. Males report modestly higher Anterior Cruciate Ligament–Return to Sport after Injury (ACL RSI) scores 9 months after ACLR, though both sexes typically exceed the proposed readiness thresholds [7]. Similarly, males show higher ACL-RSI at clearance for return-to-sport, without consistent differences in pain or kinesiophobia [8]. However, not all of the literature agrees; some report minimal or no sex differences in PROMs at mid-term follow-up [9], and others have found that change in PROMs can differ by subscale and interval, irrespective of sex [10].

Within the first three to six months after ACLR, biological healing constraints and broadly similar early rehabilitation content may limit the between-sex separation of pain, symptoms, stability, and function; however, none have examined sex-based differences in PROMs at the early time points post-surgery. Thus, the purpose of this study was to compare sex-based differences in PROMs three months after primary ACLR. We hypothesized that females would report worse values than males across all assessed PROMs at this early time point. Several scales were included in the current study, because it is an exploratory investigation conducted at an early postoperative time point following ACLR. We focused on the 3 month time point because it corresponds to an early postoperative milestone when patients typically transition from impairment-based rehabilitation toward more functional strengthening and higher-demand tasks, while biological healing constraints still limit full activity exposure.

## 2. Materials and Methods

### 2.1. Study Design and Oversight

This was a cross-sectional analysis which used data from a prospectively maintained ACL reconstruction cohort. The study was approved by the University of Delaware Institutional Review Board, and all participants provided written informed consent, with parental consent and minor assent obtained for participants younger than 18 years.

### 2.2. Participants and Setting

Fifty-six participants were assessed from a single-center longitudinal ACLR cohort. The eligibility criteria were an age of 16 to 45 years, primary unilateral ACLR, no prior lower-limb injuries or surgeries, no concurrent grade III ligament tears, and no repairable meniscal tears. The exclusion criteria were missing sex data, lack of a 3-month assessment, or incomplete PROMs.

### 2.3. Outcomes

We analyzed patient-reported outcomes expressed or transformed to a 0 to 100 percent scale at three months after surgery. The Knee Injury and Osteoarthritis Outcome Score assesses five domains that are relevant to knee disorders and ACL reconstruction populations, including symptoms, pain, activities of daily living, sport and recreation, and knee-related quality of life, and the score is validated for monitoring recovery [3]. Although several instruments are knee-focused, they were selected to reduce construct under-coverage by capturing both physical status and psychological barriers that are clinically relevant during rehabilitation. The International Knee Documentation Committee (IKDC) subjective knee form captures symptoms, knee function in daily activities, and sports participation from the patient’s perspective with strong reliability and validity across knee conditions, including ACL reconstruction [2]. The Knee Outcome Survey–Activities of Daily Living Scale (KOS ADLS) quantifies functional limitations in routine daily tasks that are attributable to knee problems [11]. The Anterior Cruciate Ligament–Return to Sport after Injury (ACL RSI) scale measures the psychological readiness for return to sport, encompassing emotion, confidence, and risk appraisal related to the reconstructed knee [12]. The Tampa Scale of Kinesiophobia quantifies fear of movement or reinjury that may limit activity and recovery [13]. All patient-reported outcome measures were converted to a 0–100 scale to facilitate comparison across instruments with different native score ranges and to allow for a uniform interpretation. Higher scores indicated a better status for KOOS, IKDC, KOS ADLS, and ACL RSI, while higher scores indicated a worse status for the TSK percentage. For interpretability, KOOS, IKDC, KOS-ADLS, and ACL-RSI were treated as better-is-higher, whereas TSK was retained as worse-is-higher (higher values indicate greater kinesiophobia). While 0–100 scaling facilitates cross-instrument comparison, it may obscure native score properties and assume linear interpretability across instruments; therefore, results should be interpreted in conjunction with instrument-specific context.

### 2.4. Statistical Analysis

Analyses were performed in IBM SPSS Statistics version 27. Data were reviewed for completeness and coding consistency prior to analysis. Primary inference used two-tailed testing at an alpha of 0.05. The normality of each outcome was assessed within sex, using the Shapiro–Wilk test. If both groups were approximately normal, independent sample *t*-tests were used with Levene’s test to check variance equality and Welch’s t was used when variances were unequal. If either group was non-normal, differences were evaluated using the Mann–Whitney U test. Effect sizes were characterized via Cohen’s d for *t*-tests and rank-based r for Mann–Whitney U tests to aid interpretability [14]. We acknowledge that post hoc power analyses do not substitute for an a priori sample size calculation and should be interpreted cautiously; therefore, we emphasize the effect sizes and confidence intervals alongside the *p* values. This approach emphasizes estimation with appropriate tests and avoids over-interpretation of *p*-values in isolation [15,16].

Post hoc power analyses were conducted using the observed sample sizes, group means or medians, and established minimal clinically important differences (MCIDs) for each outcome measure. These analyses indicated sufficient power (≥80%) to detect clinically meaningful differences for KOOS QOL, KOOS Pain, KOOS Symptoms, IKDC, and KOS-ADLS. However, the study was underpowered for TSK, KOOS Sports/Rec, ACL-RSI, and KOOS ADL, and conclusions regarding group differences for these measures should be interpreted with caution. Several outcomes had limited statistical power to detect MCID-sized differences (Supplementary Table S1), and findings for these measures should be interpreted cautiously.

## 3. Results

### 3.1. Cohort

Fifty-six participants were analyzed at approximately three months after ACL reconstruction. Females (n = 23) and males (n = 33) were similar in age, body mass index, and time from surgery. Demographic characteristics by sex are summarized in Table 1.

### 3.2. Distribution Checks

Variables considered approximately normal in both sexes were as follows: TSK, KOOS Sport/Rec, KOOS QOL, ACL-RSI, and IKDC. Variables in which at least one sex deviated from normality were as follows: KOOS Symptom, KOOS Pain, KOOS ADL, and KOS-ADLS.

### 3.3. Primary Sex Comparisons

#### 3.3.1. Parametric Outcomes

Results for the five normally distributed patient-reported outcome measures are summarized in Table 2. Across all patient-reported outcomes, no statistically significant sex-based differences were found (*p* > 0.05 for all). The effect sizes were small-to-moderate (Cohen’s d range = 0.02–0.51), indicating minimal practical differences between females and males at three months after surgery (Table 2). No statistically significant sex-based differences were detected.

#### 3.3.2. Non-Parametric Outcomes

There were no significant sex-based differences in the four non-normally distributed PROMs (KOOS Symptoms, KOOS Pain, KOOS ADL, and KOS-ADLS). Effect sizes, expressed as rank-biserial r (positive favors male, negative favors female), were small across all non-parametric outcomes, supporting the finding of minimal sex-related differences in these measures (Table 2).

In an adjusted sensitivity analysis, we modeled PROM scores as a function of sex while controlling for age, body mass index, time from surgery, and graft type. Adjusted estimates were consistent with unadjusted comparisons, and sex was not a statistically significant predictor for the evaluated outcomes (Supplementary Table S2).

## 4. Discussion

This study examined whether patient-reported outcome measures differ by sex three months after anterior cruciate ligament reconstruction. We found no statistically significant differences between females and males across a variety of PROMs, including KOOS subscales, IKDC, KOS ADLS, ACL RSI, or TSK, at this early postoperative time point. Although point estimates tended to be lower in females across several measures, these differences were not statistically significant and should be interpreted as numerical trends only. Taken together, these results do not support our hypothesis that females would report worse patient-reported outcomes compared to males three months after ACLR. Although point estimates favored males for KOOS Sport and Recreation and IKDC, these differences did not reach statistical significance and confidence intervals were compatible with small effects; therefore, these numerical trends should be interpreted cautiously and not overemphasized.

These results align with what is known about the early postoperative phase. During the first 12 to 16 weeks, biological healing and criterion-based rehabilitation constrain loading and task exposure that is similar for most patients, which likely compresses variability and limits the emergence of sex-based differences [17]. In contrast, mid-to-long term follow-ups have reported small advantages for males on knee function and sport related subscales, and modestly higher psychological readiness for return to sport [4,5,6,7,8]. Because this analysis is cross-sectional, we cannot evaluate within-person recovery trajectories; references to later divergence are based on prior longitudinal studies, rather than the present dataset. Notably, others have shown little-to-no sex-based differences at some follow-ups, reinforcing the notion that, when they occur, they are usually small and not uniform across outcomes or time points [9]. Early after ACLR, biological healing and criterion-based progression constrain activity exposure similarly across patients, which may limit detectable sex differences in perceived function. Small later differences reported in some cohorts may reflect sex-related differences in strength recovery, activity exposure, or psychological readiness as rehabilitation advances to higher-demand tasks.

While statistical insights are important, they do not necessarily reflect clinical meaningfulness. Minimally important change thresholds for measures like KOOS or IKDC have been developed; however, these are geared towards tracking participant improvement and are generally not intended for cross-sectional, group-level differences. Despite this, these estimates may serve as a reference for the purpose of framing the clinical relevance of the between-sex differences observed in this study. Although the observed KOOS Sport and Recreation mean difference exceeded the published MCID estimates, this comparison should be interpreted as exploratory because the between-sex difference was not statistically significant, observed precision was limited, and the KOOS Sport and Recreation was underpowered for MCID-sized differences (Supplementary Table S1). Accordingly, this result does not establish a sex-based difference at three months; rather, it represents an exploratory numerical trend. PROM-specific MCIDs and post hoc power estimates are provided in Supplementary Table S1 to contextualize the clinical relevance and the risk of Type II error across outcomes. This finding provides partial support for our hypothesis that females report worse outcomes early after ACL reconstruction. Males reporting higher scores may reflect early advantages in baseline strength, faster quadriceps recovery, or psychological factors such as self-efficacy and confidence with rehabilitation tasks. Additionally, the observed difference may be partially explained by sex-based variation in psychological readiness. However, the measures used to assess psychological readiness in this study (TSK and ACL-RSI) were underpowered, limiting our ability to fully interpret their contribution to the observed trends. In contrast, the IKDC minimal important change values were larger than our observed between-sex differences at three months [18], suggesting no meaningful difference between the sexes at this time point.

From a clinical standpoint, these findings support a standardized approach to early postoperative education and rehabilitation targets for both sexes. Comparable levels of pain, symptoms, daily function, sport function, knee-related quality of life, psychological readiness, and fear of movement suggest that sex alone should not dictate early rehabilitation strategies. Instead, clinicians should monitor each patient’s response and adapt progression as individuals transition to more demanding activities, where biological, psychological, or behavioral differences may begin to influence outcomes. Tracking patient-reported outcome trajectories by sex from early postoperative stages through return-to-sport decision points can provide valuable insight into how recovery unfolds over time. Integrating such longitudinal monitoring into clinical practice would help to identify when sex-specific rehabilitation strategies might be most beneficial and for whom they should be optimized.

There are several limitations that should be noted. First, the single-center cohort and modest sample size reduces the generalizability of these findings to the broader ACLR population. A larger, multi-center study may be necessary to fully characterize the influence of sex and other variables of interest on these patient-reported outcomes. Second, the results of this work are secondary to the primary outcomes of the cohort study, and thus some of the comparisons made here have been underpowered, particularly for the TSK, KOOS Sports/Rec, ACL-RSI, and KOOS ADL, as shown in Supplementary Table S1. Therefore, these findings should be considered cautiously, as the lack of statistical significance found here should not be read as definitive proof that no sex-based differences in these measures exist at this time point. Third, potential confounding variables, like graft choice, baseline activity, and rehabilitation exposure, were not controlled in this study despite their likely influence on the outcome measures assessed. Finally, the cross-sectional snapshot at three months cannot speak to trajectories or later milestones where sex-based differences could emerge. Despite this, there is a growing body of evidence to suggest that these measures may differ between sexes at post-return to sport time points and later. Collectively, these observations reinforce the importance of consistent early management coupled with ongoing evaluation as patients progress to higher functional and sport-specific demands, as well as integrating objective performance metrics and activity exposure, to fully characterize potential sex disparities and how they evolve over time. Future studies should integrate both functional performance testing and patient-reported outcomes to determine whether, and for whom, sex-specific rehabilitation strategies are warranted. Combining objective measures with psychological and subjective recovery data will provide the insight needed to optimize individualized, evidence-based rehabilitation approaches. Because this analysis is cross-sectional at 3 months, it cannot evaluate recovery trajectories over time. Unmeasured factors such as rehabilitation exposure, baseline psychosocial status, and graft-specific rehabilitation differences may have influenced PROM responses.

## 5. Conclusions

This study compared multiple patient-reported outcome measures (PROMs) between females and males three months after ACLR. No statistically significant sex-based differences were detected for any PROM, including KOOS subscales, IKDC, KOS-ADLS, ACL-RSI, and TSK. While the study was sufficiently powered to rule out a clinically meaningful difference for several key measures (KOOS QOL, KOOS Pain, KOOS Symptoms, IKDC, and KOS-ADLS), the findings for TSK, KOOS Sports/Rec, ACL-RSI, and KOOS-ADL should be interpreted with caution due to insufficient power. These findings support standardized early care while encouraging careful monitoring as patients advance to higher demand activities.

## Figures and Tables

**Table 1:** Demographic characteristics by sex.

Variable	Mean (SD)		*p* Value
	Female (n = 23)	Male (n = 33)	
Age (years)	21 (6)	24 (7)	0.09
BMI (kg/m^2^)	24.4 (4.6)	26.3 (4.1)	0.12
Time after surgery (months)	3.2 (0.3)	3.2 (0.6)	1.0
Graft type	3 Allograft	8 Allograft	0.48
	13 BPTB	16 BPTB	
	6 Hamstring	9 Hamstring	
	1 Hybrid	0 Hybrid	

*Continuous variables compared using Welch independent-sample t-tests; graft type compared using chi-square test. BPTB = bone–patellar tendon–bone*.

**Table 2:** Sex-based comparisons of percentage-scaled PROMs at 3 months after ACLR.

Outcome	Female	Male	Test Statistic	*p* Value	Effect Size
TSK	30.0 (15.6)	29.2 (17.2)	t = 0.19 (df = 53) ᵃ	0.848 ᵃ	d = 0.05
KOOS Sport/Rec	49.6 (25.5)	62.4 (25.2)	t = −1.80 (df = 53) ᵃ	0.077 ᵃ	d = 0.51
KOOS QOL	49.7 (19.7)	50.0 (17.7)	t = −0.05 (df = 53) ᵃ	0.958 ᵃ	d = 0.02
ACL-RSI	54.7 (26.9)	58.9 (23.1)	t = −0.62 (df = 53) ᵃ	0.538 ᵃ	d = 0.17
IKDC	60.2 (12.8)	66.2 (15.6)	t = −1.53 (df = 53) ᵃ	0.133 ᵃ	d = 0.42
IKDC	60.2 (12.8)	60.2 (12.8)	U = 347 ᵇ	0.592 ᵇ	r = 0.07
IKDC	60.2 (12.8)	60.2 (12.8)	U = 296.5 ᵇ	0.167 ᵇ	r = 0.13
IKDC	60.2 (12.8)	60.2 (12.8)	U = 306.5 ᵇ	0.222 ᵇ	r = 0.01
IKDC	60.2 (12.8)	60.2 (12.8)	U = 327.5 ᵇ	0.390 ᵇ	r = 0.08

*Values are mean (SD) for outcomes analyzed with independent-sample t-tests and median (IQR) for outcomes analyzed with Mann–Whitney U tests. ᵃ Independent-sample t-test. ᵇ Mann–Whitney U test. df = degrees of freedom. All outcomes are on a 0–100 scale; higher scores indicate better status, except TSK, where higher scores indicate worse status.* *Abbreviations: KOOS = Knee Injury and Osteoarthritis Outcome Score; IKDC = International Knee Documentation Committee; ACL-RSI = Anterior Cruciate Ligament Return to Sport after Injury; TSK = Tampa Scale of Kinesiophobia; d = Cohen’s d; and r = rank-biserial effect size.*

## Data Availability

The datasets generated and analyzed during the current study are available from the corresponding author upon reasonable request.

## Supplementary Materials

The following supporting information can be downloaded at: https://www.mdpi.com/article/10.3390/jcm15020680/s1, Table S1. Post-hoc Power Estimates and Minimal Clinically Important Differences (MCIDs) for Patient-Reported Outcome Measures at 3 Months Post-ACLR. Table S2. Adjusted sex association with percentage-scaled PROMs at 3-months after ACLR.
