# Supplementary material for: Sex-Based Differences in Patient-Reported Outcome Measures Are Not Present Three Months After ACL Reconstruction
Source: J Clin Med. 2026 Jan 14;15(2):680. doi: 10.3390/jcm15020680 (PMC12841943; doi:10.3390/jcm15020680)
Supplement: Supplementary file 1 [file jcm-15-00680-s001.zip › Supplementary Table S1 .pdf]

Supplementary Table S1. Post-hoc Power Estimates and Minimal Clinically Important Differences (MCIDs) for Patient-Reported Outcome Measures at 3 Months Post-ACLR

Outcome	MCID	POWER
KOOS Symptoms	15	93%
KOOS Pain	10	95%
KOOS ADL	15	12%
KOS-ADLS	10	95%
KOOS Sport/Rec	11	42%
KOOS QOL	13	96%
TSK	10	68%
ACL-RSI	15	58%
IKDC	10-16	88%
