# Supplementary material for: Sex-Based Differences in Patient-Reported Outcome Measures Are Not Present Three Months After ACL Reconstruction
Source: J Clin Med. 2026 Jan 14;15(2):680. doi: 10.3390/jcm15020680 (PMC12841943; doi:10.3390/jcm15020680)
Supplement: Supplementary file 1 [file jcm-15-00680-s001.zip › Supplementary Table S2.pdf]

Supplementary Table S2. Adjusted sex association with percentage-scaled PROMs at 3-months after ACLR.

Outcome	n	Adjusted mean Women	Adjusted mean Men	Adjusted difference (W–M)	95% CI	p value	Partial eta squared
Tampa Scale of Kinesiophobia (TSK)	56	22.6	23.7	−1.1	−4.1 to 1.9	.470	.011
KOOS Sport and Recreation (KOOS Sport/Rec)	56	52.3	65.3	−13.0	−27.9 to 1.8	.084	.061
KOOS Quality of Life (KOOS QoL)	56	47.1	46.5	0.6	−10.4 to 11.6	.912	.000
Anterior Cruciate Ligament Return to Sport after Injury (ACL-RSI)	56	51.1	53.6	−2.5	−16.9 to 12.0	.733	.002
International Knee Documentation Committee subjective score (IKDC)	56	62.0	68.8	−6.8	−15.4 to 1.9	.122	.049
KOOS Symptoms	56	73.8	76.6	−2.8	−11.7 to 6.1	.527	.008
KOOS Pain	56	81.5	86.1	−4.6	−11.6 to 2.4	.190	.035
KOOS Activities of Daily Living (KOOS ADL)	56	89.3	90.3	−1.0	−6.6 to 4.7	.740	.002
Knee Outcome Survey Activities of Daily Living Scale (KOS-ADLS)	56	82.1	85.2	−3.1	−11.4 to 5.1	.450	.012
All outcomes are on a 0–100 scale; higher indicates better status except TSK (higher indicates worse status). Adjusted means are estimated marginal means from models including age, BMI, time from surgery, and graft type.							
